# Study on the mechanism of peanut resistance to *Fusarium oxysporum* infection induced by *Bacillus thuringiensis* TG5

**DOI:** 10.3389/fmicb.2023.1251660

**Published:** 2024-04-24

**Authors:** Hongbo Du, Chuanrong Li

**Affiliations:** ^1^Forestry College, Shandong Agricultural University, Tai'an, Shandong, China; ^2^Taishan Forest Ecosystem Research Station/Key Laboratory of National Forestry and Grassland Administration for Silviculture of the Lower Yellow River, Tai'an, Shandong, China

**Keywords:** peanut, *Bacillus thuringiensis*, rhizosphere metabolite, antibiotic, carbendazim

## Abstract

Peanut *root rot*, commonly referred to as *rat tail* or *root rot*, is caused by a range of *Fusarium* species. A strain of bacteria (named TG5) was isolated from crop rhizosphere soil in Mount Taishan, Shandong Province, China, through whole genome sequencing that TG5 was identified as *Bacillus thuringiensis*, which can specifically produce chloramphenicol, bacitracin, clarithromycin, lichen VK_21_A_1_ and bacitracin, with good biological control potential. Based on liquid chromatography tandem mass spectrometry metabonomics analysis and transcriptome conjoint analysis, the mechanism of TG5 and carbendazim inducing peanut plants to resist *F. oxysporum* stress was studied. In general, for peanut *root rot* caused by *F. oxysporum*, *B. thuringiensis* TG5 has greater advantages than carbendazim and is environmentally friendly. These findings provide new insights for peanut crop genetics and breeding, and for microbial pesticides to replace traditional highly toxic and highly polluting chemical pesticides. Based on the current background of agricultural green cycle and sustainable development, it has significant practical significance and broad application prospects.

## 1 Introduction

Peanut (*Arachis hypogaea* Linn.) is an economically important legume crop with a global planting area of 26.64 million hectares and an annual production of 44 million tons (Yang and Chunlei, 2021). Peanut *root rot*, which is commonly referred to as *rat tail* or *root rot*, occurs in most peanut growing region and is one of the important factors affecting peanut yield. Generally, the incidence of this disease among plants is less than 10%, reducing production by between 5 and 8% (Ahmad et al., 2019). However, in severe cases, incidence rates can reach up to 20 to 30%, reducing production by more than 20% (Rajeswari, 2019; Anzuay et al., 2021). Between 55 and 85% of peanut *root rot* (Chen et al., 2022) is caused by a series of pathogenic microorganisms, including *Aspergillus niger* (Sharma et al., 2012), *A. flavus* (Zhao et al., 2019), *Sclerotinia* spp. (Rajeswari and Kapoor, 2017), and *Fusarium* spp. (Sun et al., 2020). The prevention and control of peanut *root rot* mainly entails the application of cultivation measures, chemical control, and biological control. Given that cultural control is generally ineffective, whereas chemical control is potentially highly toxic and leaves polluting residues, green and efficient biological control methods are currently attracting increasing attention. In contrast to other chemically synthesized fungicides, *B. subtilis* biocontrol strain *R4D2* better colonized peanut roots, reduce the occurrence of *S. rolfsii*, *A. niger,* and *Ralstonia solanarearum*, can more consistently increase peanut production, it is safer for the human body and the environment (Le et al., 2018; Ahmad et al., 2019). Due to the characteristics of good prevention control effect, low cost, and environmental friendliness without pollution, PGPR gradually becoming an important method for the prevention and control of peanut soil borne diseases.

Microorganisms, particularly bacteria, are abundant in soil, with average concentrations of 10^11^ cells per gram of soil (Mendes et al., 2013; Bhattarai et al., 2015), in which they play major roles in biogeochemical processes and have potential biotechnological contributions to plant nutrient cycling, plant growth improvement, and nutrient absorption (Yadav et al., 2021). For example, soil application of *Pseudomonas fluorescens* has been shown to reduced soil-borne *root rot* by 80.67% and contribute to increases in seedling stand, plant height, and fresh weight. *B. pumilus* have been demonstrated to stimulate increases in the biochemical parameters and antioxidant activity of peanut, and also alleviate the damage caused to peanut by *S. rolfsii* (Navya et al., 2015; Paramasivan et al., 2022), and the antagonistic strain LHSB1 of *B. velezensis* has been found to inhibit the radial growth of peanut powdery mildew hyphae by up to 93.8% (Chen et al., 2022). Some *Bacillus*, especially highly efficient PGPR with biological control functions, colonize peanut plants and induce that to resist various pathogenic microbial stresses, which is worth further research and exploration.

On the basis of their location and proximity to plants, microorganisms can be divided into different types (Edwards et al., 2015). The rhizosphere microdomain of plants is an area of concentrated microbial occurrence, succession, and metabolic activity. Rhizosphere microorganisms directly or indirectly benefit plants by providing supplementary nutrients, producing beneficial chemicals, and inhibiting pathogen growth. For example, *B. velezensis* and *B. siamensis* can inhibit the occurrence of peanut *S. rolfsii* Sacc. by producing protease, cellulase, amylase and ferroportin, and reduce the peanut disease index (Xu et al., 2020). *B. amylotica LX-J1* and *41B-1* can colonize peanut roots and induce defense responses in peanut plants, root irrigation treatment showed significant resistance to *S. rolfsii*.

Rhizosphere microorganisms respond to plant secretions and contribute to promoting plant growth and development via diverse mechanisms, including defense against pathogens (Mendes et al., 2013). The activities of total phenols, *o*-bisphenols, peroxidase, and polyphenol oxidase have been observed to increase during the different stages of *root rot* infection (Basavaraju and Devi, 2012), and consequently, gaining an understanding the rhizosphere microbial community is important for the promotion of sustainable agriculture. The rhizosphere microbial community is dynamic population that changes in response to diverse internal and external factors, thereby making it an extremely complex ecosystem (Edwards et al., 2015; Qu et al., 2020; Hinsu et al., 2021). In this regard, PGPR can significantly inhibit aflatoxin enrichment and production in inoculated plants. Differences in biochemical constituents can significantly influence the assembly of microbial communities and the activation of functional genes and metabolic pathways, as well as reducing the enrichment of aflatoxin-producing fungi, and also contributes to the manipulation of changes in peanut rhizosphere related microbiota groups (Yao et al., 2021). It is necessary to conduct in-depth research on the changes in peanut rhizosphere metabolites and related microbial populations, and explore the role of rhizosphere metabolites in peanut plants’ resistance to pathogenic microbial stress.

At present, the research of *Bacillus* has attracted great attention. *B. thuringiensis* has been popularized and applied for more than 10 years, but *B. thuringiensis* (Bt) is only used to kill insects, such as the commercial control of *Spodoptera frugiperda* (Torres et al., 2022). At present, there is no relevant report on the research of *B. thuringiensis* inducing plant disease resistance. The research and development of a microbial preparation not only has biocontrol and inhibitory effects on *F. oxysporum*, the pathogen of peanut *root rot*, but also has broad-spectrum insecticidal compound effects. It has important economic benefits for promoting the popularization and application of microbial pesticides, and has important practical significance for restoring and promoting regional water and soil resources protection and ecological diversity restoration.

## 2 Materials and methods

### 2.1 Isolation of bacterial isolates

Bacterial isolates were obtained from the rhizospheres of shrubs and grasses in the arid and barren mountains of Mount Tai, Shandong Province, China. Briefly, 5 g of rhizosphere soil was incubated with 45 mL of sterile distilled water with shaking at 220 rpm for 30 min. As a screening medium, we used Luria–Bertani (LB) solid medium, which was inoculated with isolate fermentation broth along 1% liquid inorganic phosphorus screening medium containing calcium phosphate as the sole phosphorus source. After shaking at 37°C and 180 rpm for 5 days, samples were obtained and centrifuged at 4000 rpm for 10 min, with the supernatants being collected to determine the concentration of soluble phosphorus using molybdenum antimony antibody colorimetry (Hongbo et al., 2022).

### 2.2 Physiological and biochemical identification of strains

Following Bergey’s Handbook of Bacterial Identification (Rice et al., 1995) and Microbiological Experiment Tutorial (Navya et al., 2015), the strains were subjected to gram and spore staining, and 1H-Indole-3-acetic acid (3-IAA), methyl red, voges–proskauer, gelatinase, amylase, phenylalanine dehydrogenase, hydrogenase, urease, proteolytic enzyme, and β-galactosidase tests to establish physiological and biochemical characteristics.

### 2.3 High-throughput whole-genome sequencing

Logarithmic-phase cells were harvested by centrifugation and ground with liquid nitrogen. Cell preparations were solubilized by boiling in a buffer containing sodium dodecyl sulfate, proteinase K, and mercaptoethanol, and thereafter cooled to room temperature and centrifuged. The resulting supernatant was extracted twice with the addition of chloroform/isoamyl alcohol (24: 1), with DNA being precipitated by isopropanol, followed by gentle inversion, mixing, and centrifugation. The resulting supernatant was decanted, and the pelleted material washed twice with 75% ethanol. After column purification using Ampure XP beads, the quality of the extracted DNA was assessed based on Nanodrop, Qbit, and electrophoretic analyses, Sequencing was performed using an Illumina high-throughput sequencer (NovaSeq 6,000).

### 2.4 Colonization of peanut roots by *Bacillus thuringiensis* TG5

We selected 18 healthy and well-grown peanut seedlings, which were planted in flowerpot or culture dishes (three plants per pot/dish). Samples were collected four times at 7-day intervals. The collected plants were washed repeatedly with sterile water, and we obtained 1-g samples of the roots and stems of each plant, the surfaces of which were washed with 75% alcohol for 1.5 min, followed by three rinses with sterile water, and were then washed twice with sterile water twice. The washed tissue was then ground to a paste, to which was added 1 mL of sterile water to prepare a stock suspension, which was subsequently diluted 100, 1,000, and 10,000 times to give working solutions. Aliquots of these diluted solutions were applied to solid LB medium containing 300 μg/mL rifampicin. Following inoculation with TG5, the plates were incubated for 48 h at 30°C, with each concentration being assessed in triplicate. To evaluate root colonization, the morphology and number of bacterial colonies on each plate were recorded after 7, 14, 21, and 28 days, respectively, with pure culture colony and uninoculated blank treatments being used as controls.

### 2.5 Statistical methods for peanut diseases

Peanut *root rot* disease occurs in flower producing regions around the world, and can occur at various stages of peanut growth. In the form of chlamydospore and large and small conidium, *F. oxysporum* invades directly from the wound or epidermis of peanut plants, propagates and spreads in the vascular bundle of peanut plants, making the roots of diseased plants brown and rot, vascular bundle brown, and main roots shrink and dry rot. Peanuts can be infected with diseases after sowing and before emergence, which can cause seed rot and bud rot. During the seedling stage, they are affected, leading to *root rot* and seedling wilt. During the adult stage, they are affected, leading to *root rot*, stem base rot, and pod rot. The diseased plants exhibit stunted growth, poor growth, and yellowing of leaves, ultimately leading to the entire plant withering. In production, carbendazim has a good control effect on peanut *root rot* caused by *F. oxysporum*. In this experiment, carbendazim was selected as the chemical pesticide control.

The evaluation and grading of peanut disease index mainly observe the number of diseased spots on peanut roots and stems, with the following standards: level 0, no diseased spots; Level 1, 1–3 lesions; Level 3, with numerous lesions, accounting for 1/4–1/3 of the stem and root area; Level 5, the lesion area accounts for 1/2–3/4 of the stem and root; Level 7, with patchy lesions, burning of stems, and root necrosis (Pan et al., 2022).

The calculation formula is as follows:


$$\mathrm{Disease}\;\mathrm{index}=\sum \frac{\begin{array}{c} \mathrm{Number}\;\mathrm{of}\;\mathrm{diseased}\;\mathrm{plants}\;\mathrm{at}\;\mathrm{all}\;\mathrm{levels}\\ \times \mathrm{Number}\;\mathrm{of}\;\mathrm{disease}\;\mathrm{levels} \end{array}}{\begin{array}{c} \mathrm{Total}\;\mathrm{number}\;\mathrm{of}\;\mathrm{plants}\\ \times \mathrm{Number}\;\mathrm{of}\;\mathrm{highest}\;\mathrm{disease}\;\mathrm{levels} \end{array}}\;\times 100\%$$

### 2.6 Sampling of peanut metabolites in hydroponic culture

#### 2.6.1 Design of experiments of peanut disease resistance

Select 18 flower pots with a diameter of 22 cm, add 2,500 g of industrial sand to each pot, and plant 6 peanut seeds. Water thoroughly and manage the emergence of seedlings normally for 14 days. Three test treatments, ck (control), *B. thuringiensis* TG5 treatment and carbendazim treatment, were set up, and each treatment was repeated three times. Nine pots of peanuts were inoculated with *F. oxysporum* (25 ml of *F. oxysporum* liquid fermentation liquid was irrigated with roots), and after 7 days of normal culture, 62.5 ul of purified water was added to ck treatment group, 62.5 μl of 5 × 10^6^ CFU *B. thuringiensis* TG5 was added to three pots of TG5 treatment group, 62.5 μl of carbendazim (1:800 times dilution) was added to carbendazim treatment group. Then, take 0, 24, 72 h peanut root sampling transcriptome detection, chlorophyll activity detection (chlorophyll meter), CAT activity detection (UV colorimetry) and SOD activity detection (WST-8 method), with three replicates for each sample.

#### 2.6.2 Design of experiments of resistance of TG5 strain isolate to *Fusarium oxysporum* plate confrontation

Using TG5 liquid isolate as indicator bacteria, use punch to extract 5 mm (<2) bacterial cake and inoculate them around 2 mm away from the center of the pathogen. After 4 days of cultivation, observe whether there is any antibacterial phenomenon.

#### 2.6.3 Peanut metabolite test design

Nine pots of peanuts inoculated with *F. oxysporum* were selected, and 54 peanut plants with consistent growth were randomly selected for hydroponic treatment. Three experimental treatments, ck (control), *B. thuringiensis* TG5, and carbendazim, were set up, with 6 replicates for each treatment. Each hydroponic container was filled with 200 ml hoagland culture medium, and 3 peanut seedlings were cultured in a 30°C illuminating incubator for 7 days. Add 62.5 ul of purified water was added to ck treatment group, 62.5 μL of 5 × 10^6^ CFU *B. thuringiensis* TG5 was added to three pots of TG5 treatment group, 62.5 μL of carbendazim (1:800 times dilution) was added to carbendazim treatment group, culture for 72 h continuously, and then take samples of hydroponic metabolites.

Peanut seedlings showing good growth in hydroponic culture were selected and cultured in nutrient solution, after which the roots were washed two or three times with deionized water. Thereafter, the seedlings were placed a 1-L measuring cylinder, in which they were cultivated in deionized water for 24 h, after which, we collected the solution for analysis of root exudates. The solution was filtered and subjected to vacuum rotary evaporation at 35°C. Following the addition of methanol, the residue was washed with ultrasonic oscillation, and the resulting preparation was poured onto tinfoil, having completely volatilized the methanol, the residue, which we used as root exudate, was washed two to three times and stored at –80°C until used for further analysis.

#### 2.6.4 Determination of peanut rhizosphere metabolites based on LC–MS/MS

To 20 mg of sample, we added 200 μL of methanol, followed by ultrasonic disruption for 6 min and ultrasonic disruption in ice water for 15 min. Following centrifugation for 10 min at 12000 rpm and 4°C, 10–50 μL samples of supernatant were used for analysis. Samples were injected into a ChromCore 120°C 18 chromatographic column. The column temperature was set to 40°C, mobile phase A was 0.1% formic acid and mobile phase B was100% acetonitrile. The flow rate was set to 0.3 mL/min and each sample was analyzed for 21 min.

Mass spectrometry was performed in positive and negative ion electrospray ionization (ESI) modes for detection. The ESI source conditions were as follows: ion source gas 1: 50, ion source gas 2: 50, curtain gas: 25, source temperature: 500°C (positive ion) and 450°C (negative ion), ion spray voltage floating: 5500 V (positive ion) and 4,400 V (negative ion), time-of-flight mass spectrometry (TOF MS) scan range: 100–1,200 Da, product ion scan range: 50–1,000 Da, TOF MS scan accumulation time: 0.2 s, and product ion scan accumulation time: 0.01 s. Secondary mass spectra were obtained using information dependent acquisition and high-sensitivity mode. The de-clustering potential was ±60 V and the collision energy was 35 ± 15 eV.

#### 2.6.5 Correlation analysis of transcriptome and metabonomics in peanut plants

Conjoint analysis was conducted on the data of metabolome and transcriptome of peanut root treated with carbendazim, TG5 and ck. According to the differential metabolites and differential gene expression, the “spearman” algorithm is used to analyze the correlation heat map of the differential genes and metabolites, set the threshold value for the correlation coefficient, and draw the correlation network diagram for the strong correlation pairs. Construct O2PLS-DA models using significantly different metabolites and genes. Through this analysis, we can know whether there is a trend of co-determination model differences between the two groups of data, and find out those variables that determine the correlation between model difference trends. Draw a nine quadrant map based on the expression levels of genes and metabolites, and further screen for significantly positively or negatively correlated metabolites and genes.

### 2.7 Statistical analysis

Statistical analysis data analysis was conducted using EXCEL2003 and IBM SPSS 23 statistics. Using a 5% probability level Duncan test for one-way ANOVA, compare the average values of different treatment methods (*p* < 0.05). Real time data was analyzed for homogeneity of variance and normality using Shapiro–Wilk test, and if necessary, descriptive (*D*) tests were used to compare the average values between different treatments (*p* < 0.05). All data in this paper are (mean ± SEM).

## 3 Results

### 3.1 Identification of TG5 strain

#### 3.1.1 Bacterial isolate and inorganic phosphorus solubilization

Four different bacterial isolates (TG1, TG2, TG5, and BL3) were grown in a liquid inorganic phosphorus screening medium containing calcium phosphate as the sole phosphorus source. The inorganic phosphate-solubilization abilities of the isolated strains were and for TG1, TG2, TG5, and BL3, respectively (Supplementary Figure S1). The TG5 strain, which was characterized by the best phosphorus solubilization performance, was selected for further analysis.

#### 3.1.2 Strain colony morphology and biochemical and physiological characteristics

The TG5 strain was characterized as a gram-positive bacterium, the colonies of which are milky white, opaque, irregular, raised, and have an undulating margin. Among the physiological and biochemical tests, positive results were obtained for the 3-IAA preliminary screening and ACC (acetyl-CoA carboxylase, ACC) deaminase tests (Supplementary Table S1).

#### 3.1.3 Genome sequencing

**Figure 1:**
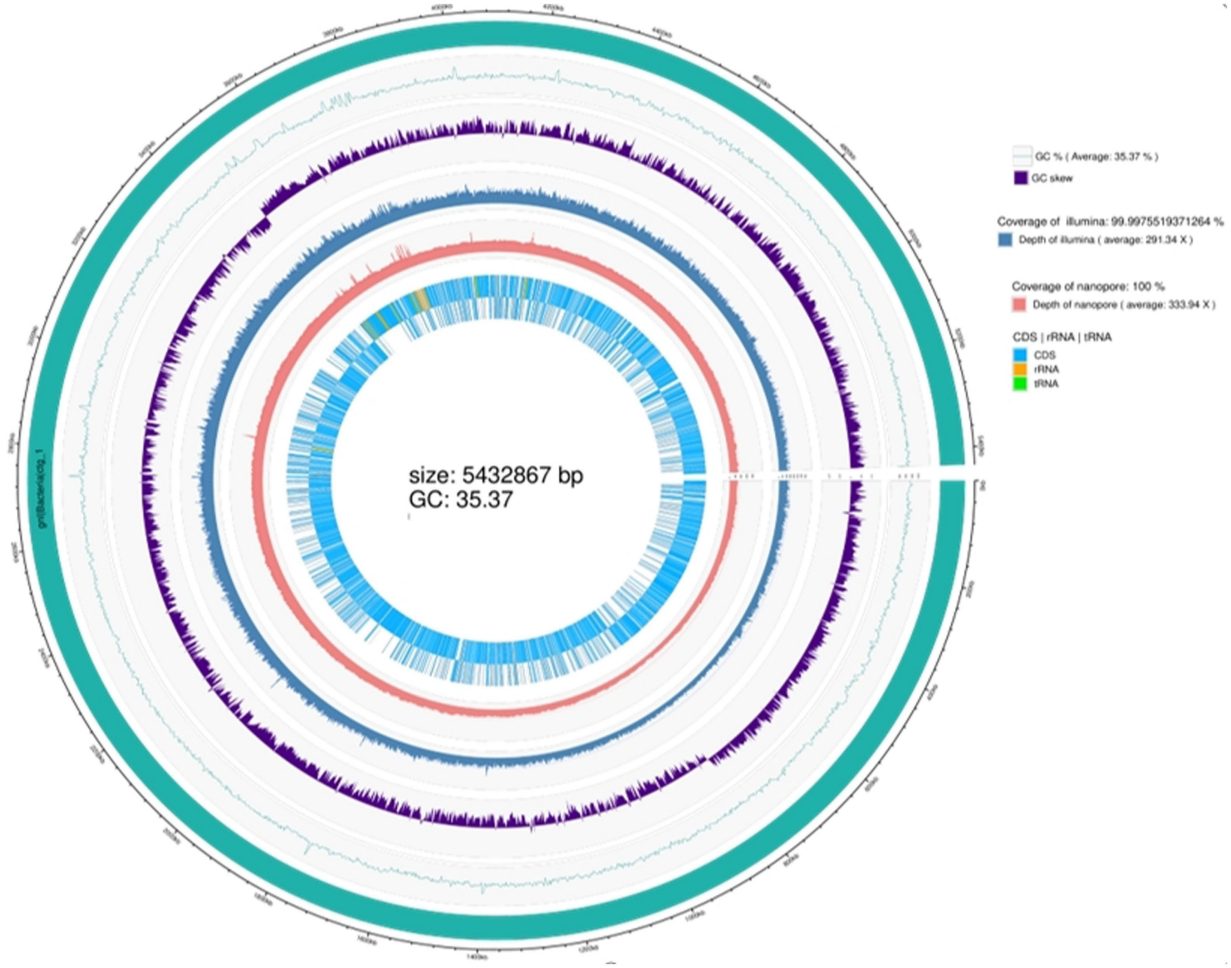
Whole genome sequence of strain TG5.

Whole-genome sequencing revealed that the genome of the TG5 strain contains a single chromosome and three plasmids with sizes of 5.34 Mbp and 40,886, 21,700, and 1,526 bp, respectively. The genome contains 6,342 coding sequences, five rRNA synthesis operons, and 355 tRNA synthesis genes (Supplementary Figures S2–S5). The strain has been registered as *B. thuringiensis* (TG5; NCBI SUB12195433, access number CP110119.1). It contains the amino acids glutamic acid, cysteine, lysine, proline, histidine, alanine, and glycine and other related metabolic genes (*glsA1*, *ddL-1*, *xerC-3*, *trcC-2*, *thiO-1*, *proS-2*, *hdc_2*, and *ald1-1*), a global nitrogen regulatory gene (*ntcA*). It also contains growth-promoting phosphorus metabolism (*sfp*) and chitinase metabolic (*chiA1*) genes (Supplementary Table S2), as well as genes associated with the synthesis of chloramphenicol, bacillicin, patulin, clarithromycin, the antibiotic VK_21_A_1_, and bacitracin (*cat_2*, *nprM_1*, *lgrD_2*, *rdmC*, *lchA1_2*, and *bceA_1*) (Supplementary Table S3), and is accordingly considered to have good biological control potential (Figure 1).

### 3.2 Changes in root and stem length and disease index of peanuts under different treatments

**Figure 2:**
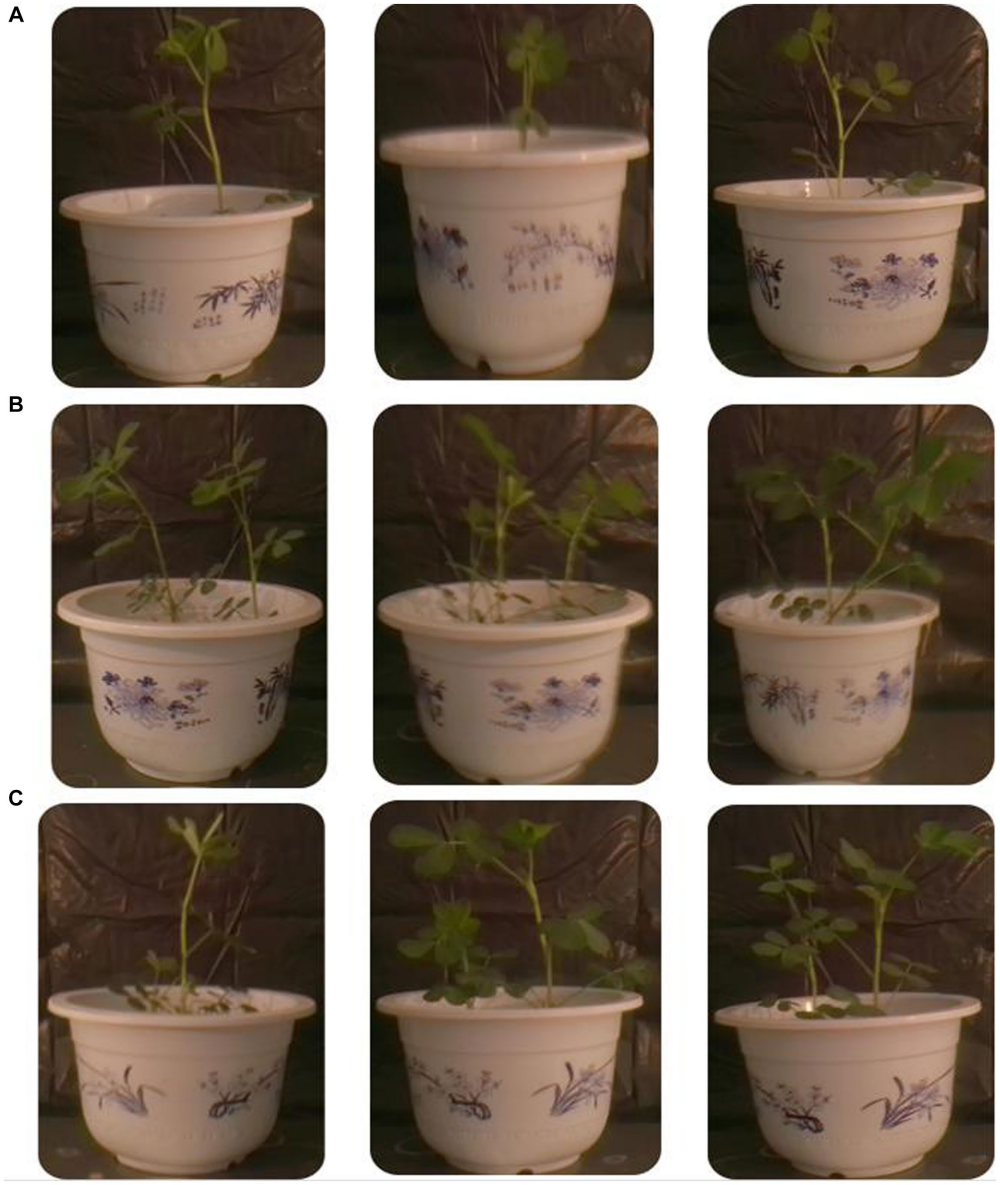
Different treatments of peanut plants. **(A)** CK treatment infected with *F. oxysporum* peanut plant. **(B)** TG5 treatment infected with *F. oxysporum* peanut plant. **(C)** Carbendazim treatment infected with *F. oxysporum* peanut plant.

**Figure 3:**
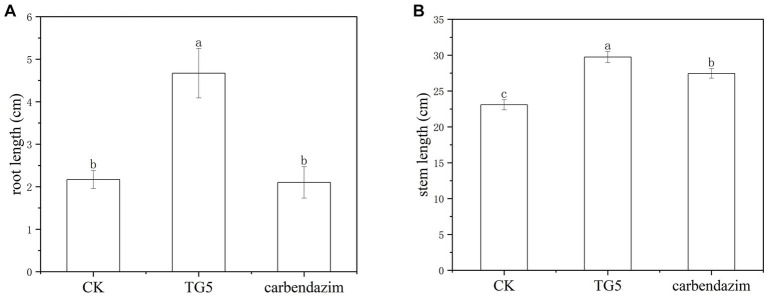
Comparison of the effects of carbendazim and *B. thuringiensis* TG5 treatment on peanut seedling growth. **(A)** Peanut root length under different treatments. **(B)** Peanut stem length under different treatments.

Peanut *root rot* disease occurs in important flower production areas in China, occurring at various growth stages of peanuts. The main symptoms are rotten roots and dead seedlings, resulting in severe seedling shortage and broken ridges, leading to the death of seedlings in patches. The TG5 and carbendazim treatment were inoculated into the roots of peanut seedlings to study the disease resistance effect of different treatments on peanut plants infected with *F. oxysporum*. Compared with the control group, carbendazim treatment had an inhibitory effect on the infection of *F. oxysporum* in peanuts, with a disease index of 20.48% for peanut root rot disease, which was hidden at level 4. Compared with the control, TG5 treatment can effectively control peanut *root rot* caused by *F. oxysporum*, and the control effect is very significant, and peanut rhizome length increased by 56.20 and 28.45%, respectively, (*p* < 0.05), which also shows that TG5 has great potential to replace carbendazim to control peanut root rot caused by *F. oxysporum* (Table 1 and Figures 2, 3).

**Table 1:** Peanut disease index under different treatments.

Disease grading	Level 1	Level 2	Level 3	Level 4	Level 5	Level 6	Level 7	Disease index 100%
CK				✱	✱✱✱	✱	✱✱	66.67
TG5	✱	✱						2.86
carbendazim				✱✱	✱	✱	✱	20.48

In the table, ✱✱ represents partial distribution, ✱✱✱ represents more than one-third distribution, and ✱✱✱✱ represents half distribution.

### 3.3 TG5 treatment and carbendazim treatment induced peanut resistance to *Fusarium oxysporum* stress

**Figure 4:**
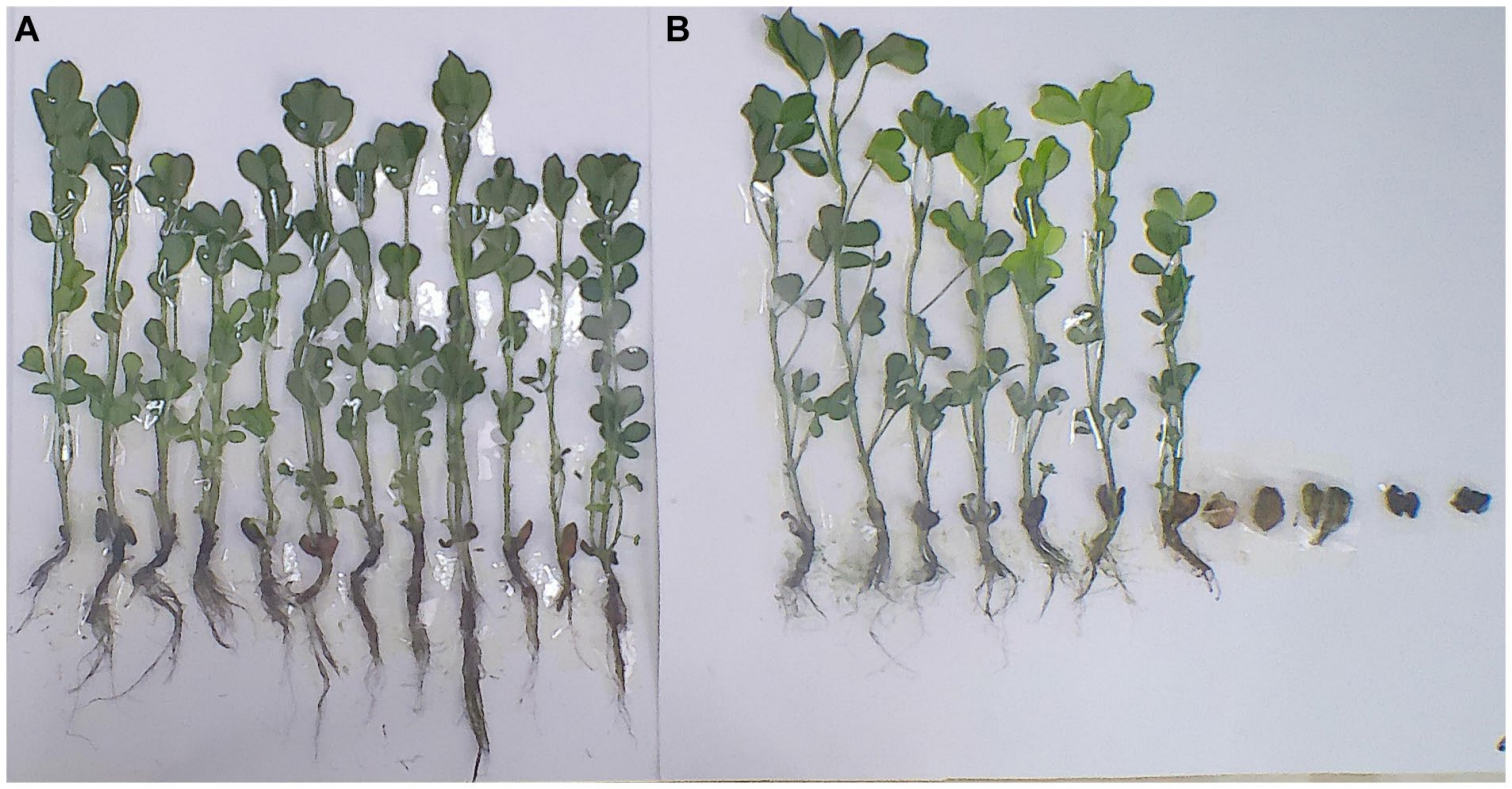
TG5 treatment and carbendazim treatment induced peanut resistance to *F. oxysporum* stress. **(A)** TG5 treatment induces peanut plants to resist *F. oxysporum* stress; **(B)** carbendazim treatment induced peanut plants to resist *F. oxysporum* stress.

The treatment of *B. thuringiensis* TG5 can induce peanut plants to resist *root rot* caused by *F. oxysporum*, and the effect is significant. Compared with carbendazim treatment, the survival rate of peanut plants increased by 71.4% (*p* < 0.05), meanwhile, peanut plants grew healthily and had developed roots, showing a good biocontrol effect (Figure 4).

### 3.4 TG5 isolate inhibits the growth of *Fusarium oxysporum*

**Figure 5:**
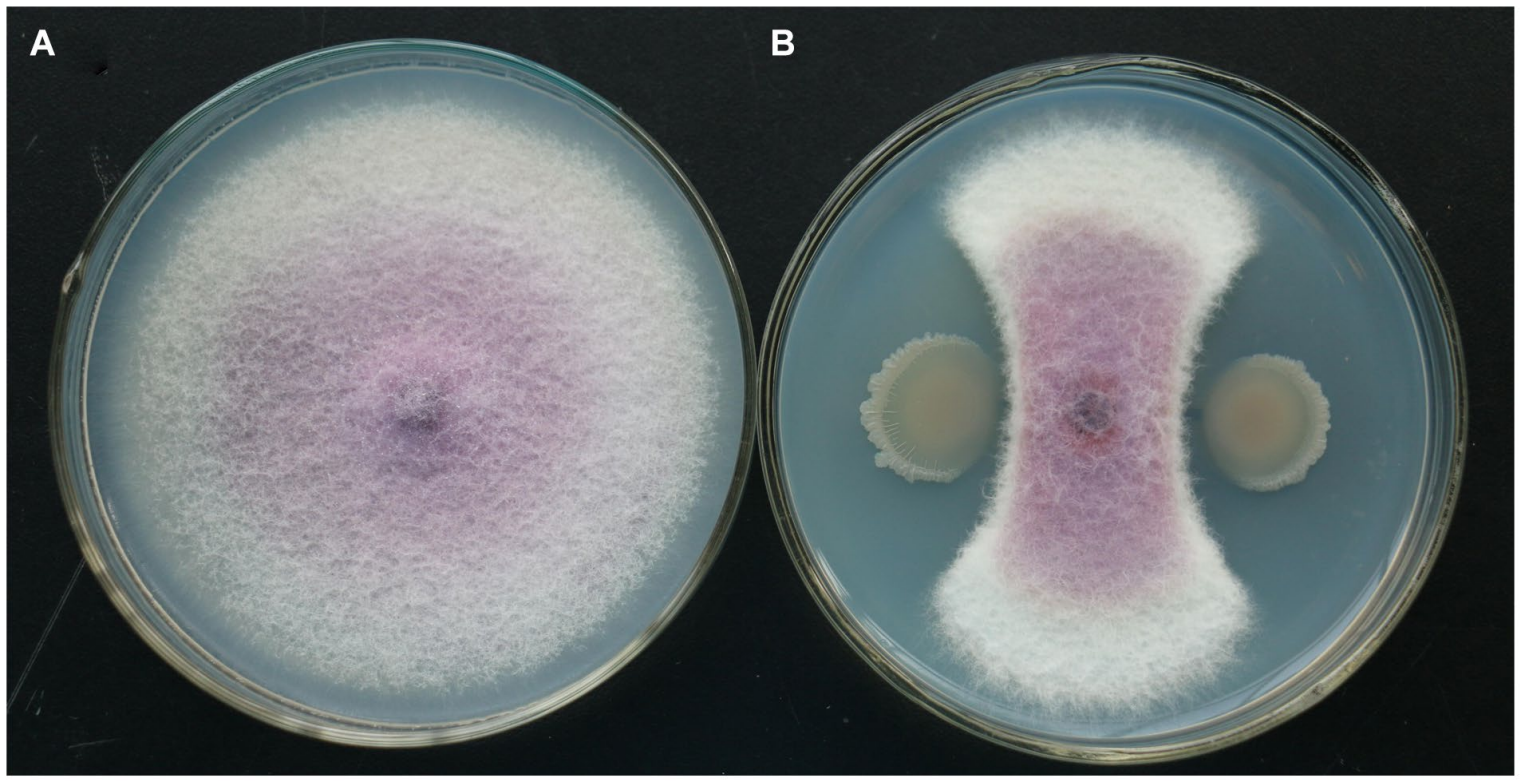
TG5 isolate inhibits the growth of *F. oxysporum.* **(A)** Control growth of *F. oxysporum*; **(B)** TG5 isolate inhibits the growth of *F. oxysporum*.

In the plate confrontation experiment, the isolate of TG5 strain had a significant inhibitory effect on *F. oxysporum*. Compared with the control, the isolate of TG5 strain inhibits the radial growth of *F. oxysporum* colonies, which are distributed in a fan-shaped pattern and form a clear antibacterial ring (Figure 5).

### 3.5 Colonization of peanut roots by *Bacillus thuringiensis*

**Figure 6:**
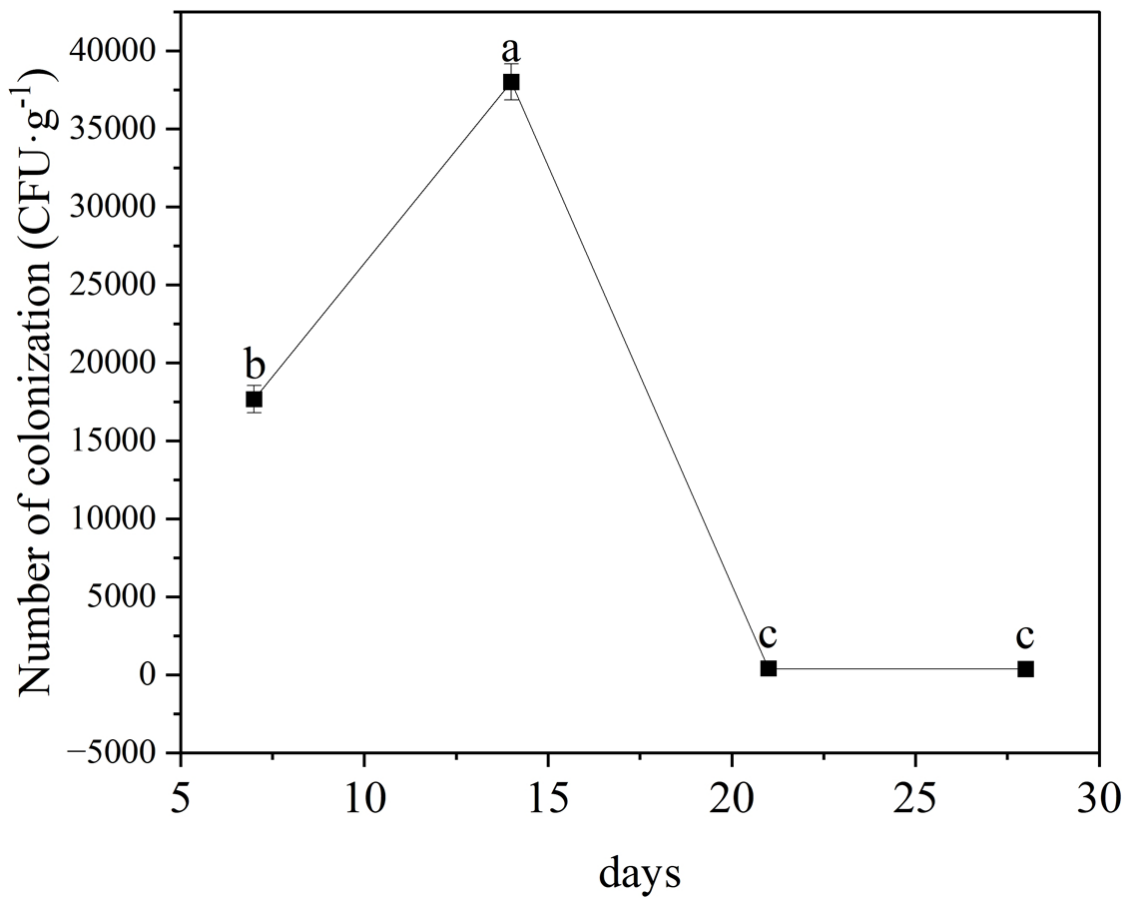
Analysis of root colonization of *B. thuringiensis* TG5 in peanut.

We found that the TG5 strain of *B. thuringiensis* can stably colonize the roots of peanut seedling, with colonization levels peaking at 3.8 × 10^4^ CFU following colonization for 14 days (Figure 6). Thereafter, levels tended to stabilize from 21 days post-inoculation.

### 3.6 Changes in chlorophyll and protective enzymes in peanuts under different treatments

**Figure 7:**
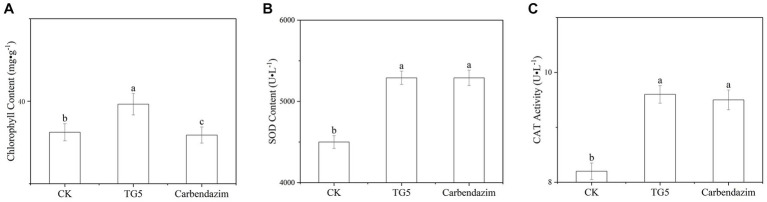
Changes in chlorophyll and protective enzymes in peanuts under different treatments. **(A)** Changes in chlorophyll content in peanuts. **(B)** Changes in SOD content in peanut plants. **(C)** Changes in CAT content in peanut plants. The letters “a, b, and c” indicate the degree of data difference between different treatments.

Chlorophyll is the main pigment that characterizes the photosynthetic capacity of plants. Compared with the control, the chlorophyll content of peanut leaves after TG5 and carbendazim treatment has changed (Figure 7). The chlorophyll content of peanut leaves after TG5 treatment is better than that of the control and carbendazim treatment. Although the change of a single leaf is not very significant, considering the number of leaves, the chlorophyll difference of the whole plant and the whole plot will be great, and the accumulation of substances caused by photosynthesis will be very surprising. The accumulation of reactive oxygen species can cause membrane lipid peroxidation and loss of membrane differential permeability in plants, leading to defense reactions such as allergic necrosis and phytoalexin production, superoxide dismutase and catalase can eliminate reactive oxygen free radicals and superoxide anion in plants, which are closely related to plant disease resistance. Compared with the control, after 72 h of TG5 treatment, the contents of superoxide dismutase and catalase in peanut plants increased by 23.2 and 14.0%, respectively, (*p* < 0.05), significantly increasing the stress resistance of peanut plants. Compared with the traditional pesticide carbendazim, the effect of TG5 treatment on superoxide dismutase and peroxidase was not significantly different, indicating that TG5 biopesticide had the potential to replace the traditional pesticide carbendazim.

### 3.7 Cluster analysis results of correlation between different treatments of peanuts

**Figure 8:**
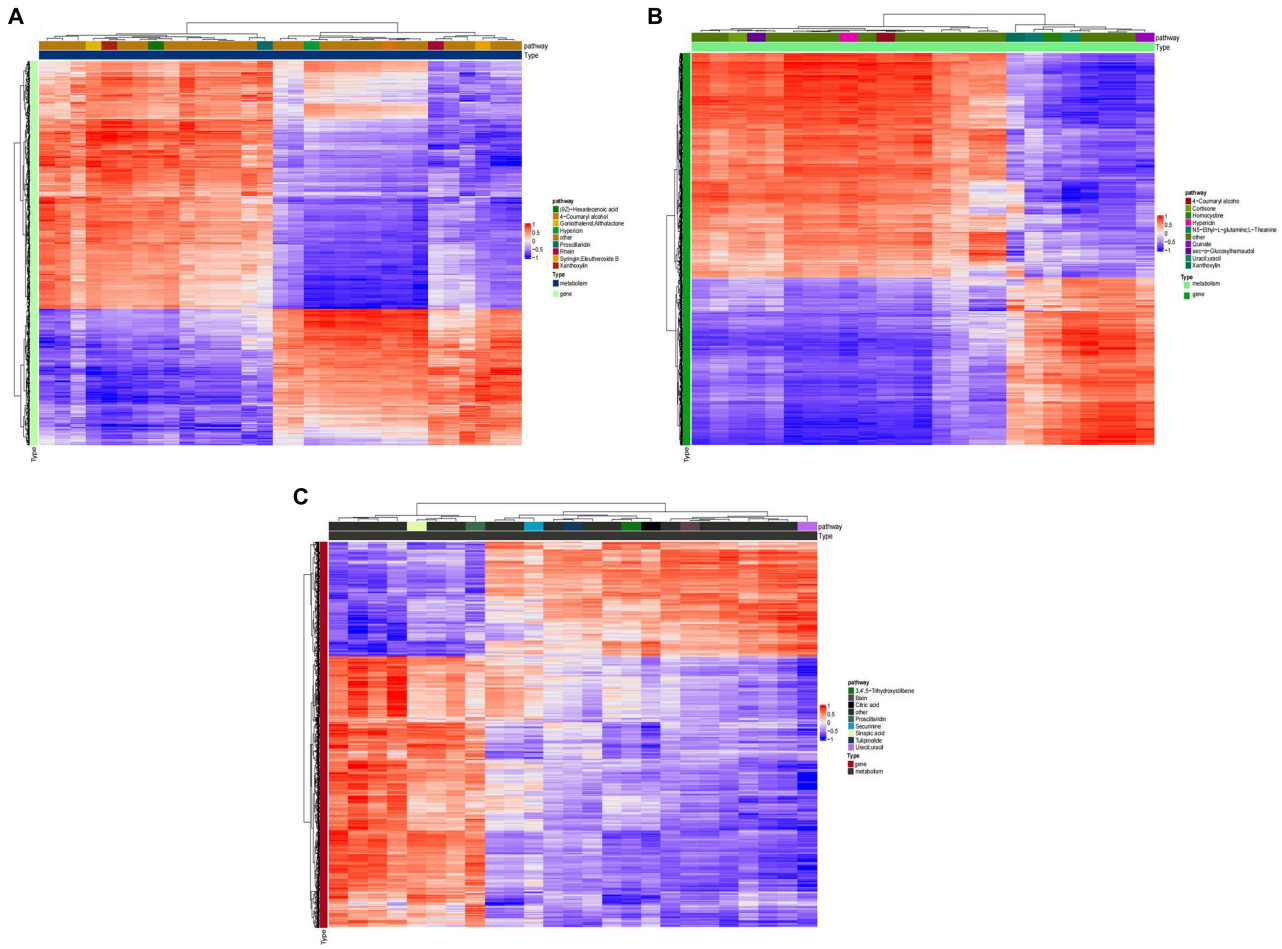
Cluster heat map of peanuts with different treatments. **(A)** Cluster analysis between TG5 and ck. **(B)** Cluster analysis between carbendazim and ck. **(C)** Cluster analysis between TG5 and carbendazim.

Figure 8 shows that, TG5 treatment has better correlation with transcriptome activity than ck treatment in 3,4‘,5−trihydroxystilbene, sinapinic acid, citric acid, and algin. These metabolites are closely related to plant resistance, that is, ck treatment may have fewer resistant substances than TG5 treatment. TG5 treatment was superior to carbendazim treatment in the correlation between coumarol, theanine, quinate, homocysteine and transcriptome activity, and it may be that the resistance of TG5 treatment may be stronger than that of carbendazim treatment.

### 3.8 Correlation network analysis of different peanut treatments

**Figure 9:**
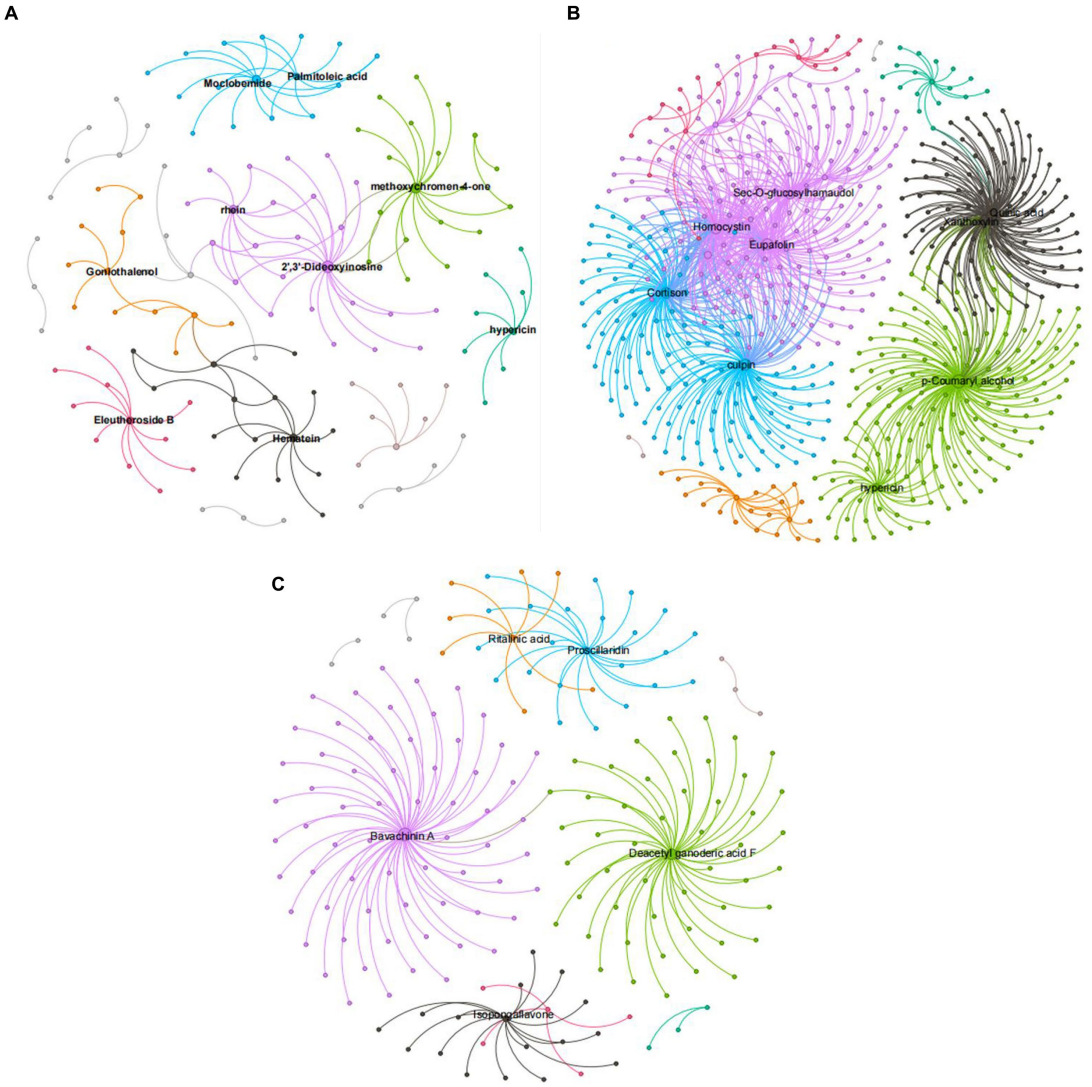
Correlation network diagram of different peanut treatments. **(A)** Correlation network diagram between TG5 treatment and ck treatment. **(B)** Correlation network diagram between carbendazim treatment and ck treatment. **(C)** Correlation network diagram between TG5 treatment and carbendazim.

Figure 9 shows that, compared with ck treatment, the metabolites of carbendazim treatment are sec-o-glucose formosanol, homocysteine, quinic acid, lignin, chlorophyll, p-coumarol, TG5 treatment is relative to ck, and the metabolites of carbendazim treatment are palmitoleic acid, moclobemide, methoxy group pigment, 2‘,3’-dideoxyinosine, carrageenan, carbendazim treatment is relative to TG5 treatment. The metabolites that are highly correlated with transcriptional genes are vitamin A, seaweed glycoside, deacetylated ganoderma lucidum F, and isoflavones. Analysis shows that these metabolites may play a direct or indirect regulatory role in key metabolism that forms different phenotypes in different treatments.

### 3.9 Screening of differential genes and metabolite correlation in peanuts

**Figure 10:**
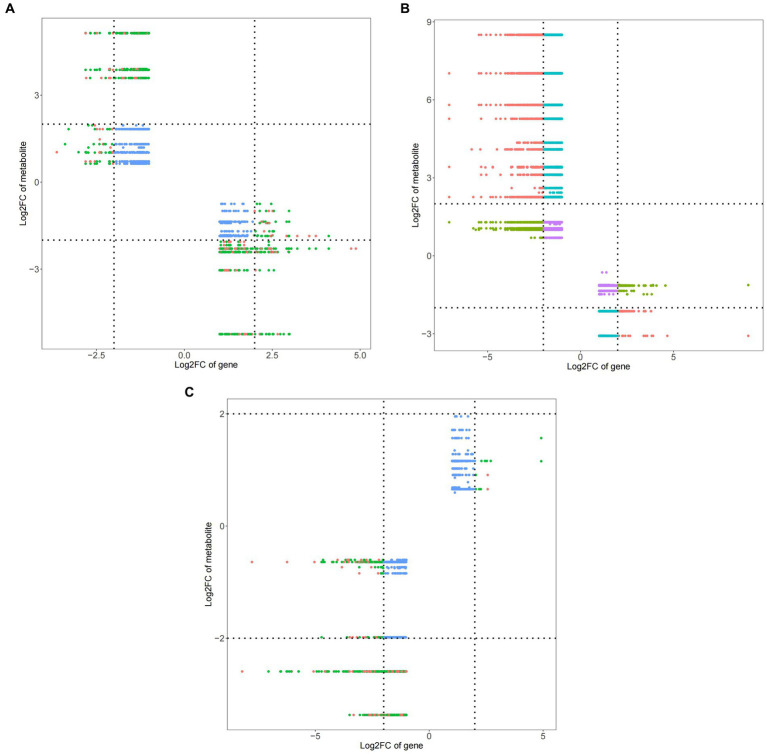
Nine quadrant diagram of peanuts with different treatments. **(A)** TG5 treatment and ck treatment nine quadrant diagram. **(B)** Carbendazim treatment and ck treatment nine quadrant diagram. **(C)** TG5 treatment and carbendazim treatment nine quadrant diagram.

Compared with ck treatment and TG5 treatment, ck and carbendazim treatments had significantly more DEMs and DEGs in quadrant 1 and quadrant 9 (Figure 10), indicating that the metabolites of carbendazim treatment changed strongly compared with TG5 treatment. Compared with TG5 treatment, carbendazim treatment had less positive and negative correlation DEMs and DEGs, indicating that the correlation between the two groups of samples was small. The relationship between phenotypic differences and metabolic differences between the two is relatively small.

Compared with ck group, carbendazim treatment had the highest correlation with the differential gene 4,8-dimethyldeca-4,8-dieonic acid, and the highest correlation with the differential metabolite was *LOC112695144*.

Compared with ck group, TG5 treatment had the highest correlation with differential genes, and the highest correlation with differential metabolites was *LOC112754465* (repetitive proline rich cell wall protein 1).

Compared with TG5 treatment, carbendazim treatment has the highest correlation with differential genes β-catechol carboxylic acid methyl ester, *LOC112754465* (repetitive proline rich cell wall protein 1) is the most relevant with differential metabolites.

### 3.10 Analysis results of O2PLS model in peanut processing room

**Figure 11:**
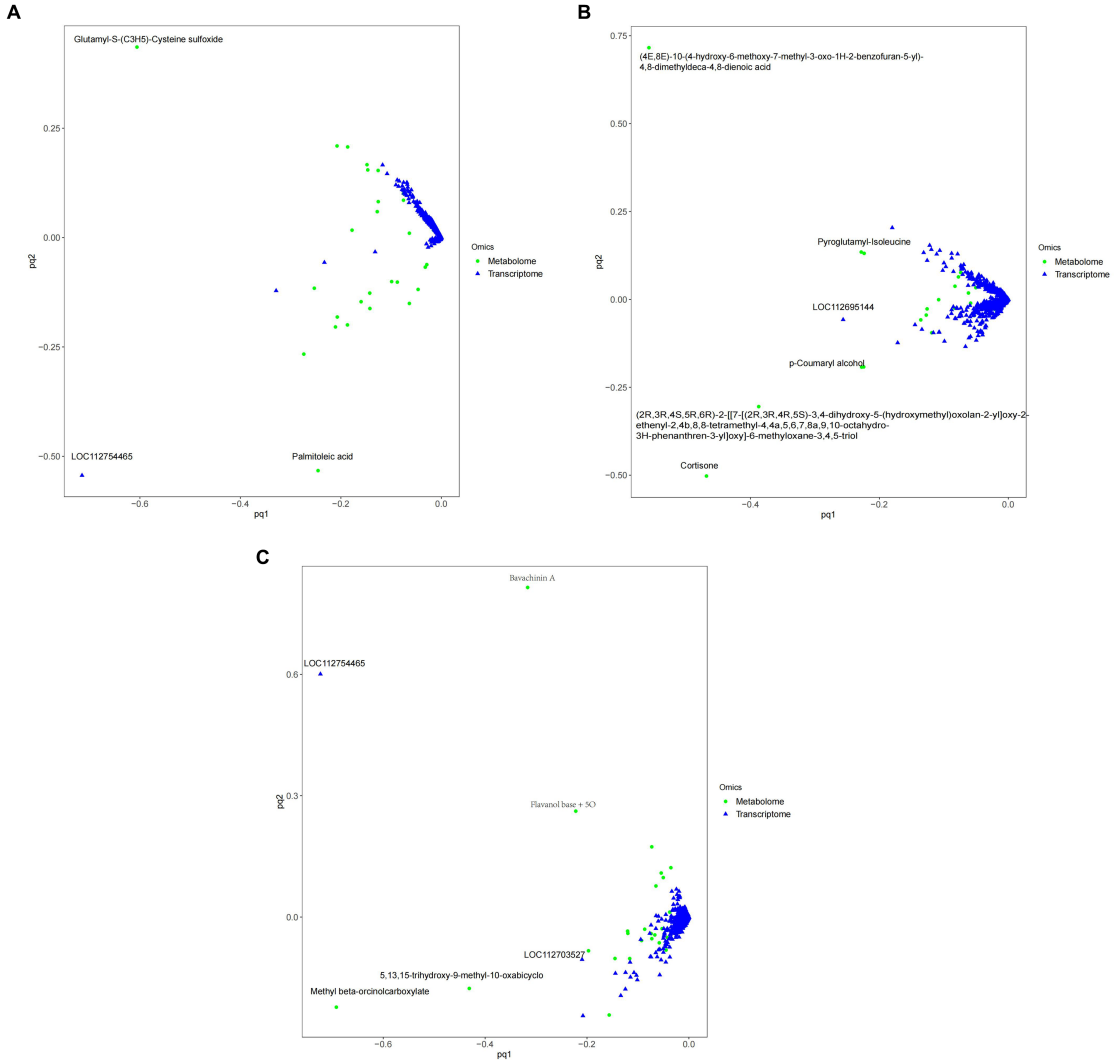
O2PLS model diagram of peanuts with different treatments. **(A)** TG5 treatment and ck treatment O2PLS model diagram. **(B)** Carbendazim treatment and ck treatment O2PLS model diagram. **(C)** TG5 treatment and carbendazim treatment O2PLS model diagram. The distance height from each point to the origin in the diagram represents the magnitude of metabolites and gene correlation.

Figure 11 shows that, Compared with ck group, carbendazim treatment had the highest correlation with the differential gene 4,8-dimethyldeca-4,8-dieonic acid, and the highest correlation with the differential metabolite was *LOC112695144*.

Compared with ck group, TG5 treatment had the highest correlation with differential genes, and the highest correlation with differential metabolites was *LOC112754465* (repetitive pro line rich cell wall protein 1).

Compared with TG5 treatment, carbendazim treatment has the highest correlation with differential genes β-4-catechol carboxylic acid methyl ester, *LOC112754465* (repetitive proline rich cell wall protein 1) has the highest correlation with differential metabolites.

### 3.11 Result of KEGG enrichment in peanut processing room

**Figure 12:**
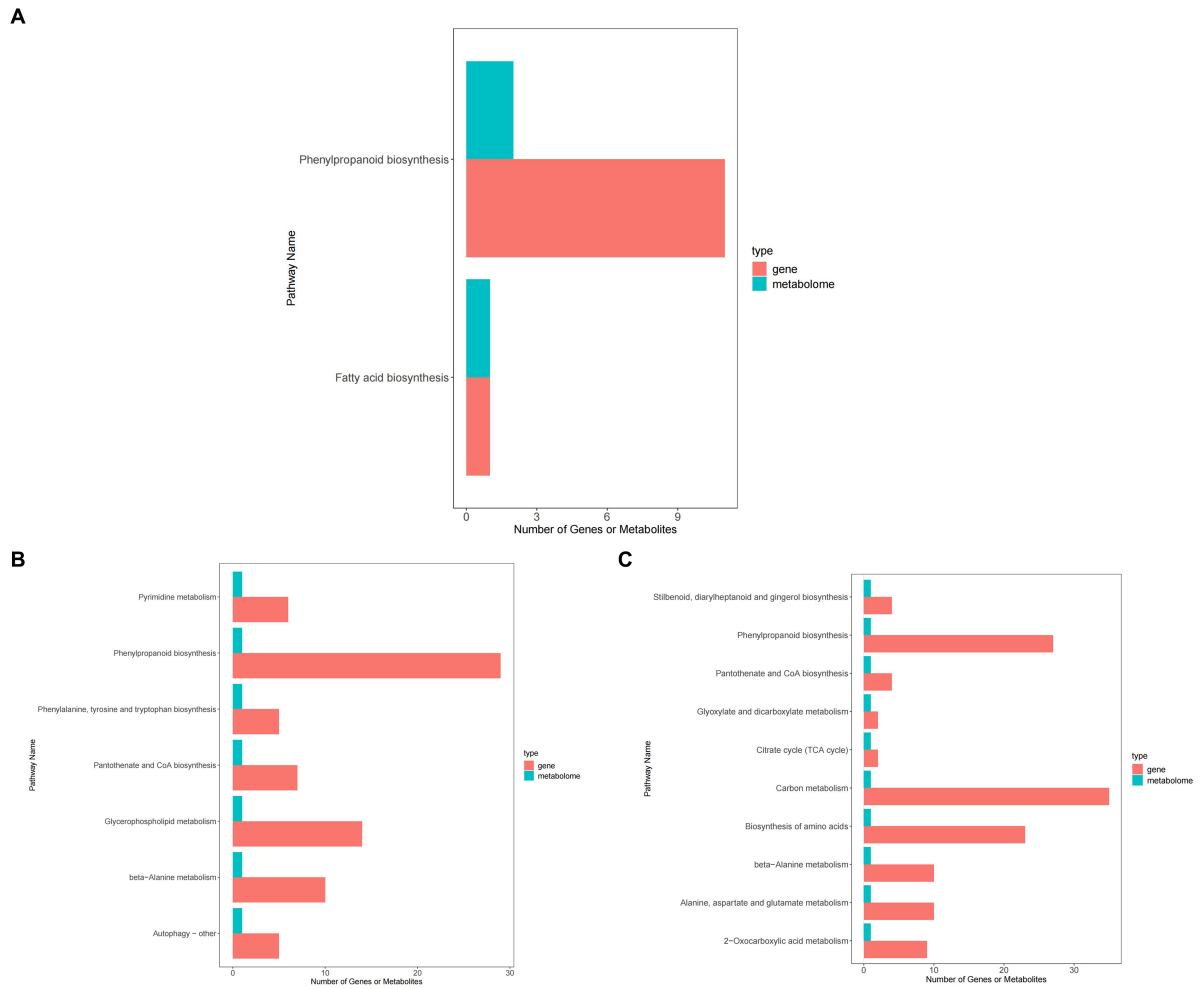
KEGG enrichment map of peanuts under different treatments. **(A)** KEGG enrichment map of TG5 treatment and ck treatment. **(B)** KEGG enrichment map of carbendazim treatment and ck treatment. **(C)** KEGG enrichment map of TG5 treatment and carbendazim treatment.

Figure 12 shows that, compared with ck treatment, carbendazim treatment mainly concentrated differential metabolites and differential genes in pyrimidine metabolism, phenylalanine biosynthesis, phenylalanine, tyrosine and tryptophan biosynthesis, pantothenic acid and COA biosynthesis, glycerophospholipid metabolism, β-alanine metabolism, autophagy related pathways. It has a certain relationship with disease resistance.

TG5 treatment compared with ck treatment, differential metabolites and differential genes were mainly enriched in phenylpropanoid biosynthesis, fatty acid synthesis related pathways, carbendazim treatment compared with TG5 treatment, differential metabolites and differential genes were mainly enriched in the biosynthesis of stilbenes, diarylheptanoids and gingerols, and 2-oxocarbonic acid metabolism, β-Alanine metabolism, pantothenic acid and COA biosynthesis, glyoxylic acid and dicarboxylic acid metabolism related pathways.

### 3.12 Analysis of peanut metabolic pathways

**Figure 13:**
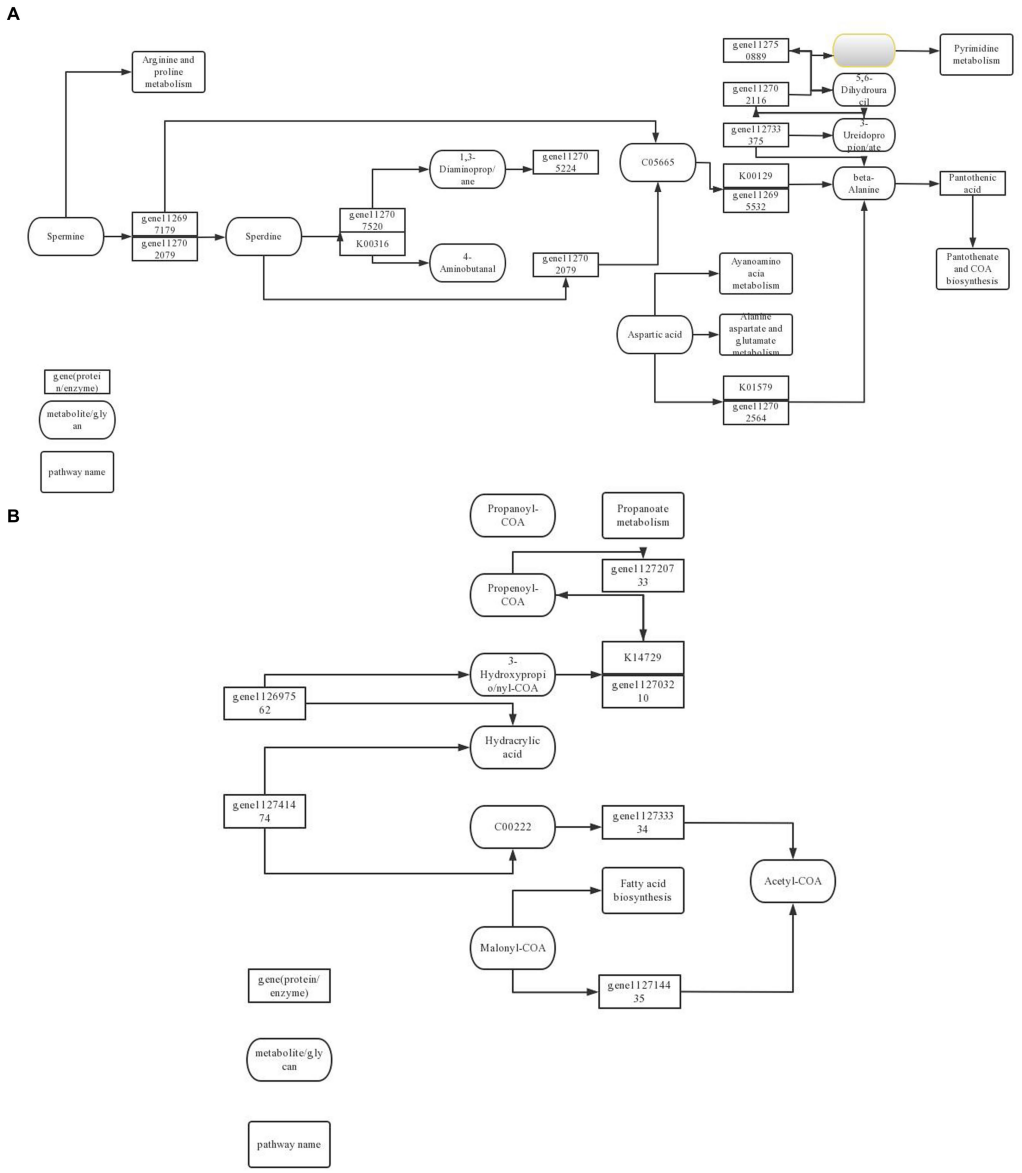
TG5 microbial fertilizer induces peanut antibacterial metabolic pathway. **(A)** Amino acid metabolism pathway; **(B)** Fatty acid biosynthesis pathway.

Cell autophagy is closely related to the initiation of cell defense response. In the comparison of KEGG metabolic pathways treated with ck and carbendazim treatment, it was found that differential genes and differential metabolites were enriched in the autophagy metabolic pathway, and the main metabolite sec-*o*-glucosylhamaudol was significantly up-regulated in the pathway, and related genes were also significantly accumulated (Figure 13).

Phenylpropanoids can be differentiated into many substances related to cell defense, such as coumarin, lignin, etc. In the comparison of KEGG metabolic pathways treated with ck and TG5 treatment, it is found that differential genes and differential metabolites are enriched in the phenylpropanoids metabolic pathway, and the main metabolites proscillaridin, rhein, etc. are significantly up-regulated in the pathway, and related genes are also significantly accumulated.

Biosynthesis of stilbenes, diarylheptanoids and gingerols are important secondary metabolite in plants, and they have a great relationship with plant defense response. In the comparison of KEGG metabolic pathway between carbendazim treatment and TG5 treatment, it was found that differential genes and differential metabolites were enriched in this pathway, the main metabolite desoximethasone was significantly up-regulated in the pathway, and related genes were also significantly accumulated.

## 4 Discussion

Different species of *Bacillus* can solubilize phosphorus and potassium, fix nitrogen, promote plant growth, and inhibit pathogenic microorganisms. In this study, we found that PGPR can significantly inhibit aflatoxin enrichment and aflatoxin production in inoculated peanut plants (Navya et al., 2015). Differences in biochemical compounds can significantly influence microbial community assembly, the activation of functional genes and metabolic pathways, the enrichment of aflatoxin-producing fungi, and the manipulation of peanut-associated microbial groups (Yao et al., 2021). Certain species of *Bacillus* have been established to have strong antagonistic effects on peanut powdery mildew (Chen et al., 2020), and *B. pumilus* has been shown to have an inhibitory effect on peanut blight in southern China (Xu et al., 2020), whereas *P. fluorescens* has been identified as a good plant biocontrol agent (Basavaraju and Devi, 2012). In this study, we found that *B. thuringiensis* TG5 liquid isolate can significantly inhibit the growth of *F. oxysporum* in the plate confrontation test, which is supported by the results of pot experiments. Inoculation of peanut seedlings with TG5 was found to promote seedling growth and changes in the concentration of multiple metabolites in the peanut rhizosphere, including squarenol, a synthetic precursor of sterols. TG5 treatment was also shown to significantly regulate the activity of superoxide dismutase and stimulate increases in the secretion of glycyl-valine, glutamyl-S-(C_3_H_5_)-cysteine sulfoxide and a rage of amino acids that can directly affect plant growth. Furthermore, we detected an increase in the production of palmitoleic acid, a low molecular weight organic acid secreted by plant roots that is essential for plant nutrition and resistance to cationic toxicity, and is core component of the response to different types of abiotic stress and the interaction between roots and soil microorganisms (Wang et al., 2021). In addition the higher concentration of organic acids (citric acid and oxalic acid) and amino acids (glutamic acid, glycine, and glutamine) detected in response to TG5 treatment can contribute to increasing the growth of *Rhizobium* and the formation of biofilms, and promote the enhancement of *Rhizobium*-mediated nodulation in peanuts (Navya et al., 2015). Among the compounds positively influenced by TG5, the feedback inhibition of heme is a key regulatory step in the synthesis of chlorophyll (Wu et al., 2008). For comparative purposes, we also examined the effects of carbendazim, a broad-spectrum bactericidal compound, on peanut growth. Following application, we detected a significant increase in the production of quinic acid, which can serve as a precursor for the biosynthesis of aromatic amino acids or as a carbon source for some soil bacteria. With respect to other PGPR, we found that *P. fluorescens*-treated peanut seeds stored for 6 months were characterized by significantly reduced levels of aflatoxin infection and aflatoxin yield. Moreover, the TG5 strain colonized in peanut seeds can vertically spread and benefit the offspring of the host plant. Therefore, the development of microbial preparations by *B. thuringiensis* TG5 can not only be used as microbial insecticides, but also can induce related plants to resist pathogenic microbial stress, which helps reduce the dependence on highly toxic pesticides in peanut production (Xie et al., 2019), and has great application and promotion value.

Plant growth promoting rhizobacteria (PGPR) can respond to plant secretions and assist plants through various effects that promote plant growth, including defense against pathogens (Mendes et al., 2013; Meena et al., 2020; Essalimi et al., 2022). Roots secrete plant specific metabolic substances with diverse biological activities and ecological functions, which affect the assembly and metabolism of plant rhizosphere microorganisms and are crucial for plant growth and health (Nakayasu et al., 2023). The change of root exudates contributes to the enhancement of peanut rhizobia nodulation (Wang et al., 2021). The root exudates planted by the endophytic fungus phyllostachys formosana significantly increased the rhizosphere bacteria abundance, soil respiration, microbial biomass and enzyme activity of long-term continuous peanut soil, promoted peanut nodulation and N_2_ fixation, and specific root exudates reduced the nitrate (NO_3_^−^) in the rhizosphere soil and increased the number of slow growing rhizobia associated with peanut nodulation and the number of bioactive strains, it is conducive to enhancing the symbiosis between peanut and slow growing rhizobia (Xie et al., 2019). Deeply studying PGPR in inducing plant resistance expression through changes in peanut rhizosphere metabolites and related gene expression is a very effective approach. This study found that TG5 treatment induced the content of 3,4‘,5-trihydroxystilbene, sinapinic acid, citric acid, algal glycoside and other metabolites closely related to plant resistance better than ck treatment. In addition, TG5 treatment induced more coumarol, theanine, quinate, homocysteine and other metabolites in peanut than carbendazim treatment, which also indicated that TG5 treatment might induce better resistance to *F. oxysporum* stress in peanut than carbendazim treatment.

After induction by *B. thuringiensis* TG5 and carbendazim, peanut plants secrete glutamyl-s-cysteine sulfoxide and 4,8-dimethyldeca-4,8-dieonic acid, respectively, as defense means. Some bioactive compounds in peanuts (Kuei et al., 2021; Park et al., 2021; Duke, 2022; Li et al., 2022), including resveratrol (Luo et al., 2021; Zhu et al., 2022), β-sitosterol, proanthocyanidin, linoleic acid and arachidonic glycosides have inhibitory effects on plant pathogenic bacteria and fungi. To further explore the metabolic pathway and mechanism of microbial pesticides inducing peanut disease resistance, and the difference in disease resistance between microbial pesticides and traditional chemical pesticide carbendazim, provide data and theoretical support for the development and promotion of microbial pesticides, which is of great practical significance for sustainable agricultural development.

PGPR strains (Kumar et al., 2021; Samuel and Oluranti, 2022; Binnian et al., 2023), especially those with highly efficient promoting (Madhusmita et al., 2021; Batoule and Risa, 2022) and biocontrol effects (Amna et al., 2020; Mukesh et al., 2020), will undoubtedly occupy a dominant position in future production practices. Meanwhile, PGPR carrier (Sang et al., 2020; Twinkle et al., 2021; Zhu et al., 2022) is one of the main factors restricting the promotion and application of microbial agents. There are two basic principles for carrier selection, firstly, it is easy to absorb and utilize the target microorganisms, promoting the increase of microbial population density and the display of various characteristics; the second is that it can be widely used in industrial production, which is common and easy to obtain, preferably other metabolic waste (Guo et al., 2020) or agricultural waste (Fatima et al., 2021; Gomez and Uribe-Velez, 2021; Le et al., 2023) at the end of the industrial chain. In addition, the addition of synergistic substances such as chitosan (Agri et al., 2021; Milica et al., 2022; Roohallah et al., 2022) and other marine products in the culture medium is receiving increasing attention; the effective colonization of microbial strains in target organisms and the compatibility of two or more complex microbial communities are other factors that restrict the promotion of microbial agents and must be given sufficient attention.

## 5 Conclusion

The plate confrontation test showed that TG5 strain could inhibit the growth of *F. oxysporum*, and the inhibition effect was obvious, and the pot experiment confirmed this result. The survival rate of peanut plants treated with TG5 was 71.4% higher than that treated with carbendazim, and the plants grew stronger, the roots were more developed, and the leaves were black and shiny.

Compared with the control, the highest correlation between TG5 treatment and the differential gene is glutamyl-s-cysteine sulfoxide, and the highest correlation with the differential metabolite is *LOC112754465* (repetitive proline rich cell wall protein 1). The KEGG metabolic pathway found that the differential gene and differential metabolite were enriched in the phenylalanine metabolic pathway, and the main metabolites proscillaridin, rhein etc., were significantly up-regulated in the pathway, TG5 treatment enriched more resistant substances to induce peanut resistance to *F. oxysporum* stress.

However, compared with the control, the highest correlation between carbendazim treatment and differential genes was 4,8-dimethyldeca-4,8-dieonic acid, and the highest correlation with differential metabolites was *LOC112695144*. The KEGG metabolic pathway found that differential genes and differential metabolites were enriched in autophagy metabolic pathway, and the main metabolite sec-o-glucosylhamaudol was significantly up-regulated in the pathway, and related genes were also significantly accumulated. Compared with TG5 treatment, carbendazim treatment has the highest correlation with differential genes β-catechol carboxylic acid methyl ester, the highest correlation with the differential metabolite is *LOC112754465*. In the KEGG metabolic pathway, it is found that the differential gene and differential metabolite are enriched in this pathway. The main metabolite desoximethasone is significantly up-regulated in the pathway, and the related genes are also significantly accumulated. The TG5 treatment induced peanut resistance to *F. oxysporum* stress may be stronger than carbendazim treatment.

## Data availability statement

The data presented in the study are deposited in the NCBI repository, accession number CP110109.1.

## Author contributions

All authors listed have made a substantial, direct, and intellectual contribution to the work and approved it for publication.
