# Supplementary figures and images for: Study on the mechanism of peanut resistance to Fusarium oxysporum infection induced by Bacillus thuringiensis TG5
Source: Front Microbiol. 2024 Apr 24;14:1251660. doi: 10.3389/fmicb.2023.1251660 (PMC11080293; doi:10.3389/fmicb.2023.1251660)

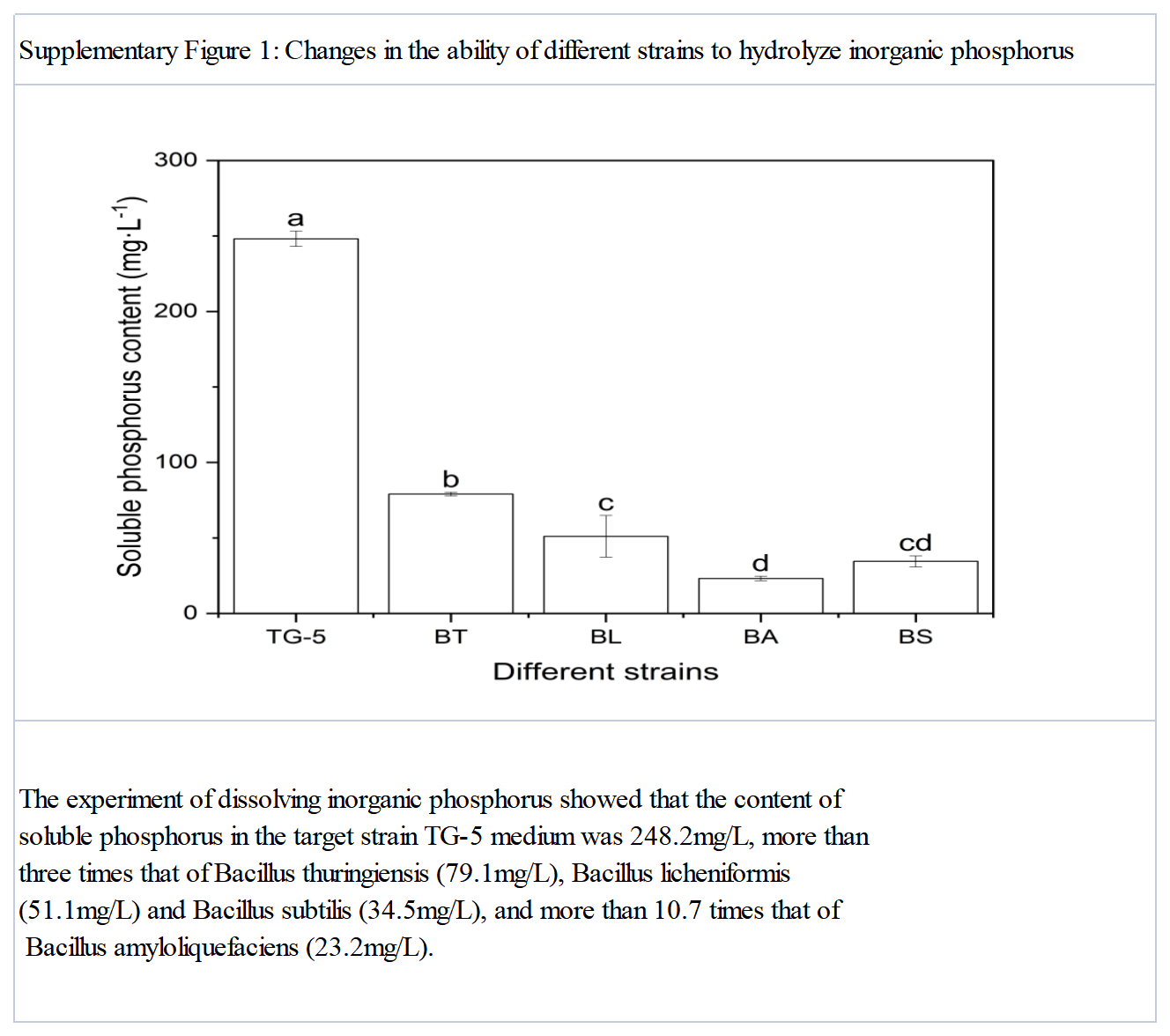

Supplement: Supplementary file 4 [file Image_1.tif]

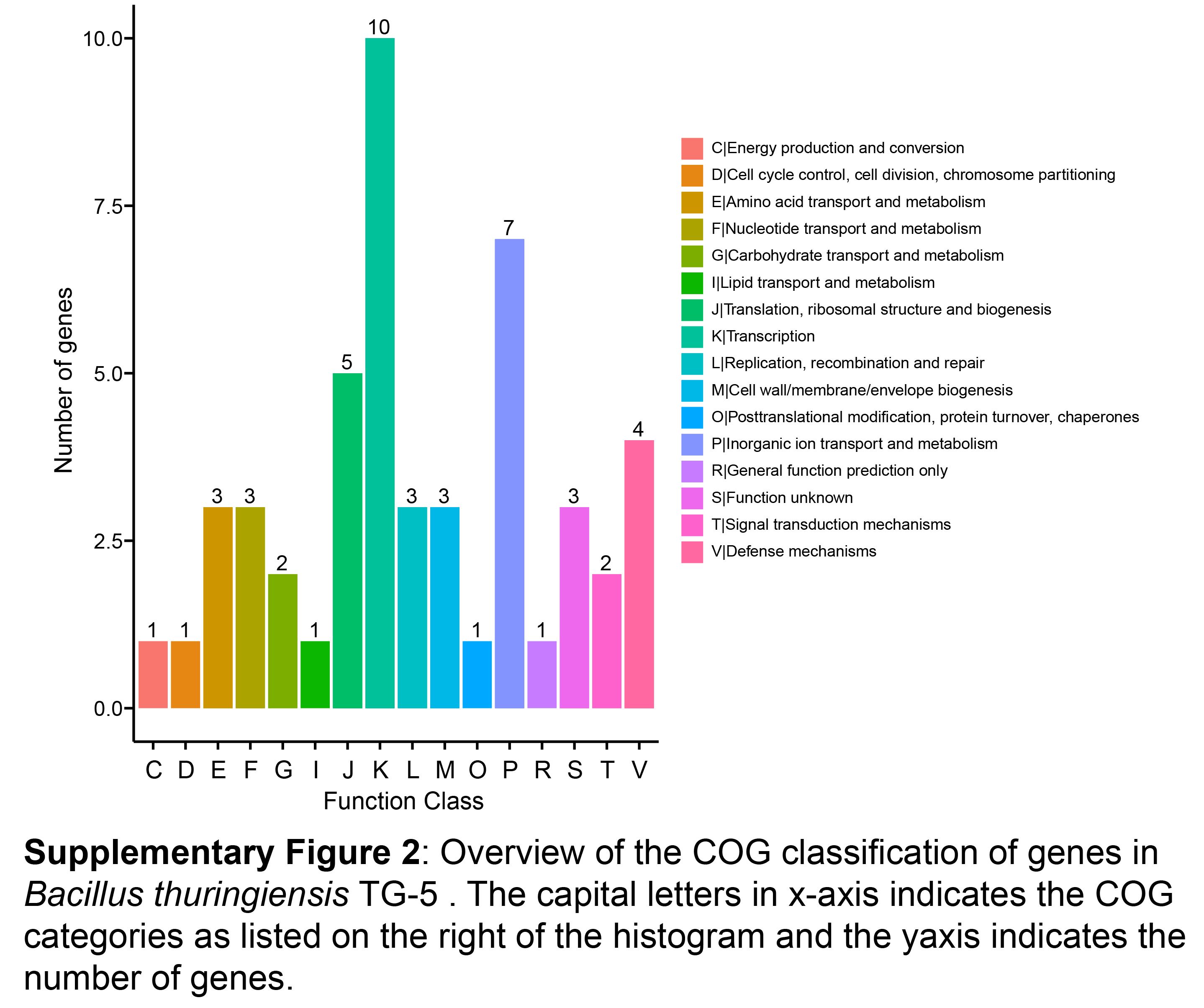

Supplement: Supplementary file 5 [file Image_2.tif]

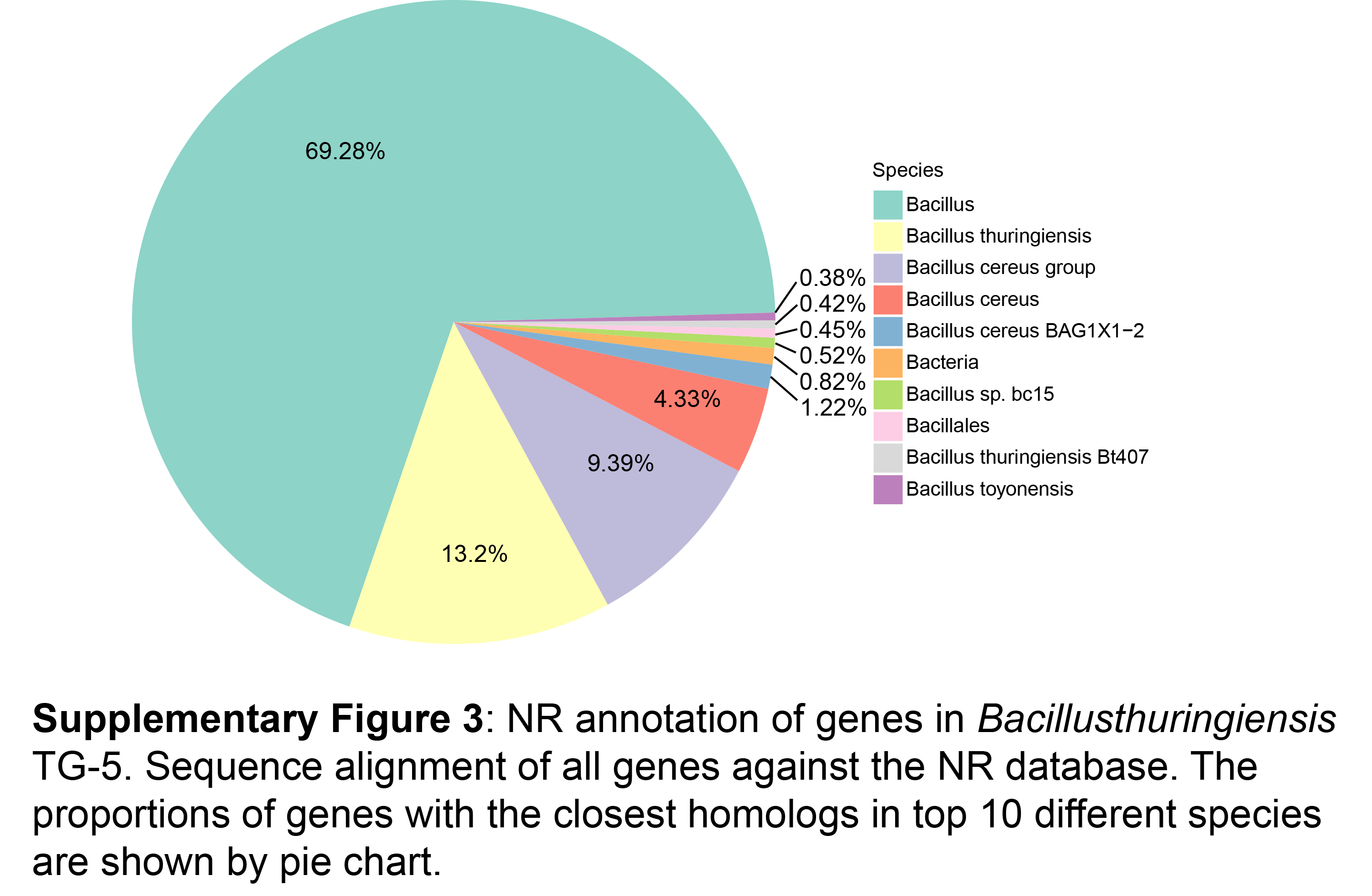

Supplement: Supplementary file 6 [file Image_3.tif]

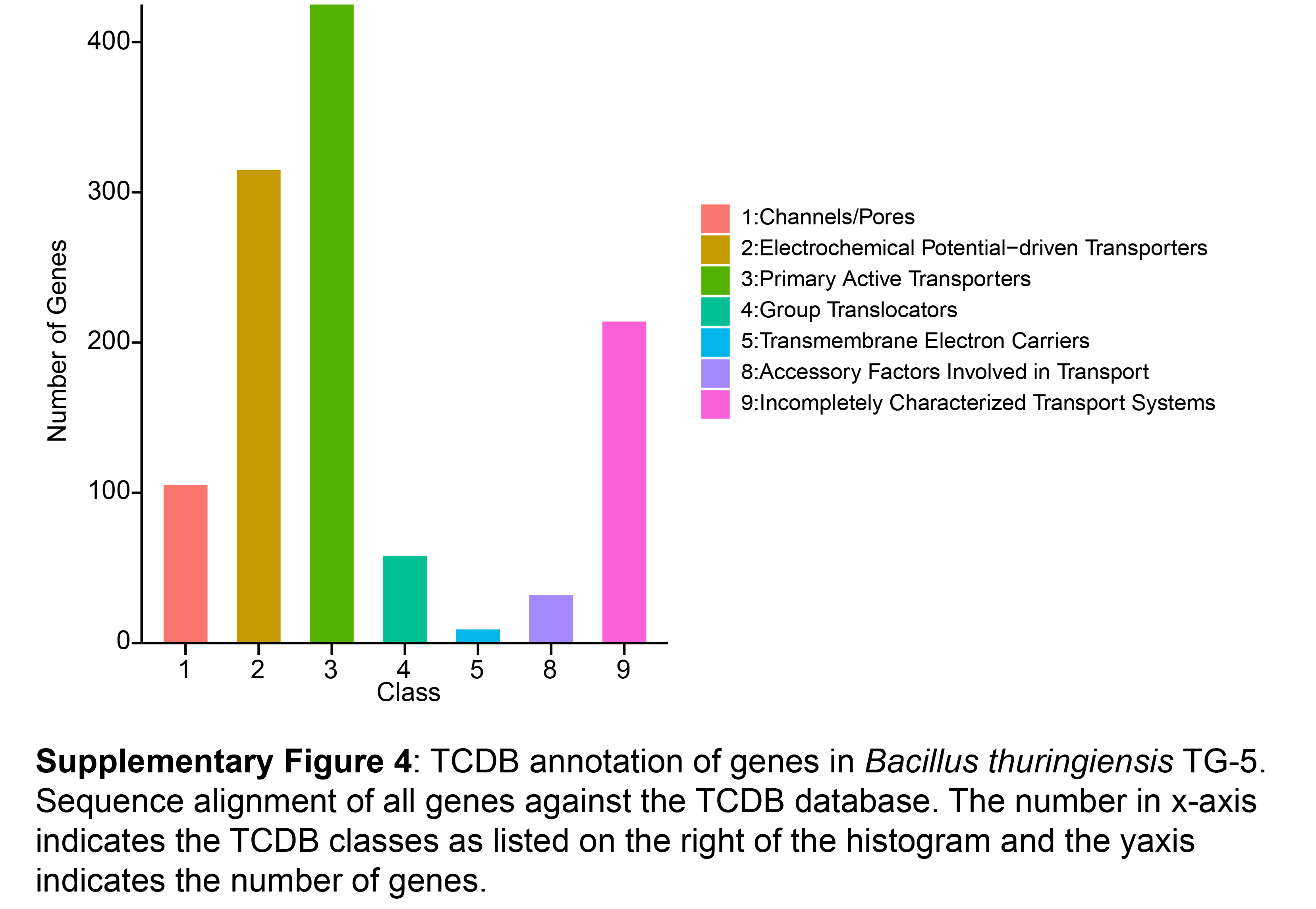

Supplement: Supplementary file 7 [file Image_4.tif]

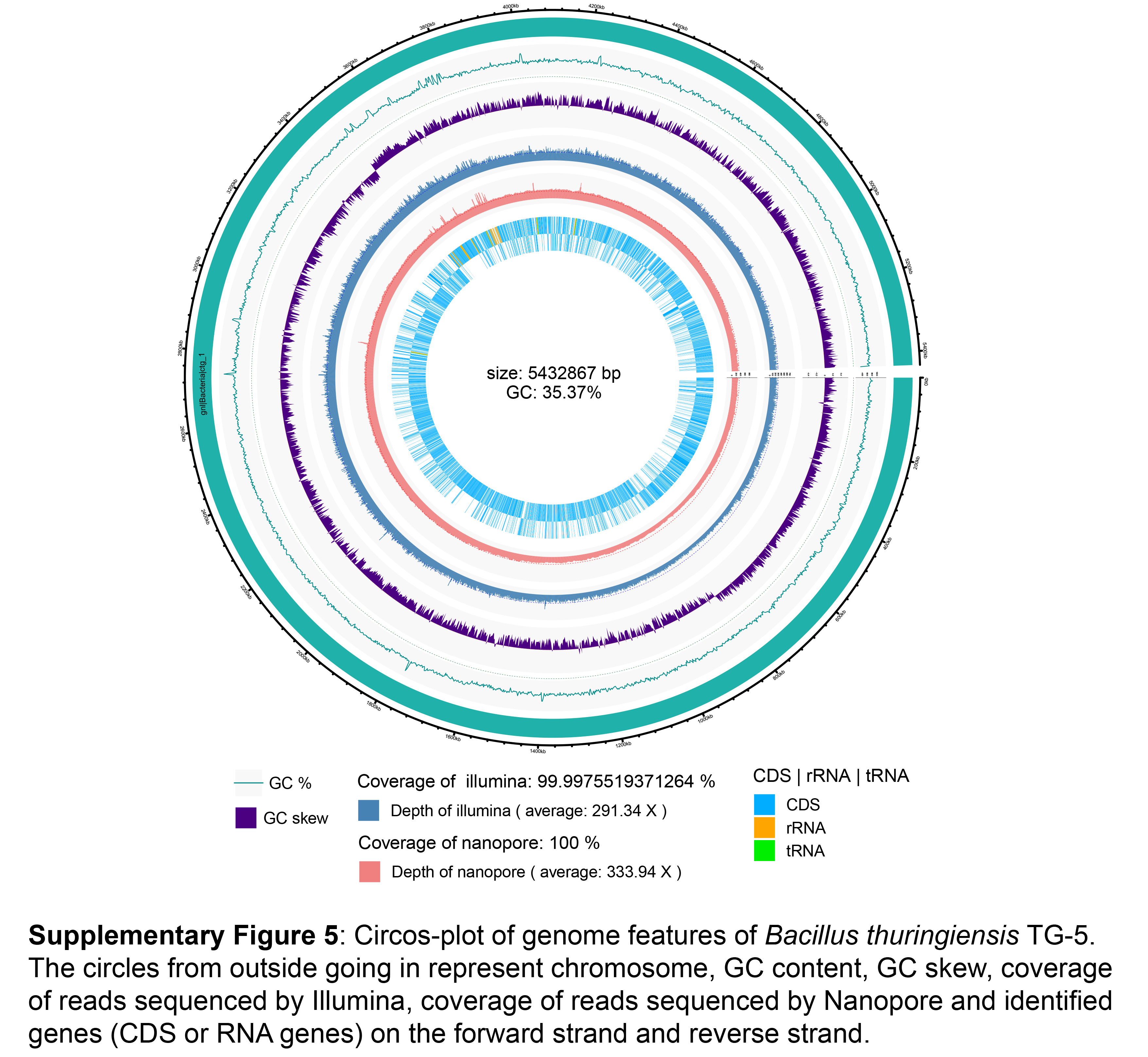

Supplement: Supplementary file 8 [file Image_5.tif]
